# Adverse events after surgery for injuries to the subaxial cervical spine: analysis of incidence and risk factors

**DOI:** 10.1007/s00068-024-02458-2

**Published:** 2024-02-16

**Authors:** Philipp Raisch, Jan Pflästerer, Michael Kreinest, Sven Y. Vetter, Paul A. Grützner, Matthias K. Jung

**Affiliations:** Clinic for Trauma and Orthopedic Surgery, BG Trauma Center Ludwigshafen, University of Heidelberg, Ludwig-Guttmann-Straße 13, 67071 Ludwigshafen On the Rhine, Germany

**Keywords:** Subaxial cervical spine injury, Surgical adverse event, Complication, Risk factor

## Abstract

**Purpose:**

To determine the incidence of severe surgical adverse events (sSAE) after surgery of patients with subaxial cervical spine injury (sCS-Fx) and to identify patient, treatment, and injury-related risk factors.

**Methods:**

Retrospective analysis of clinical and radiological data of sCS-Fx patients treated surgically between 2010 and 2020 at a single national trauma center. Baseline characteristics of demographic data, preexisting conditions, treatment, and injury morphology were extracted. Incidences of sSAEs within 60 days after surgery were analyzed. Univariate analysis and binary logistic regression for the occurrence of one or more sSAEs were performed to identify risk factors. *P*-values < .05 were considered statistically significant.

**Results:**

Two hundred and ninety-two patients were included. At least one sSAE occurred in 49 patients (16.8%). Most frequent were sSAEs of the surgical site (wound healing disorder, infection, etc.) affecting 29 patients (9.9%). Independent potential risk factors in logistic regression were higher age (OR 1.02 [1.003–1.04], *p* = .022), the presence of one or more modifiers in the AO Spine Subaxial Injury Classification (OR 2.02 [1.03–3.96], *p* = .041), and potentially unstable or unstable facet injury (OR 2.49 [1.24–4.99], *p* = .010). Other suspected risk factors were not statistically significant, among these Injury Severity Score, the need for surgery for concomitant injuries, the primary injury type according to AO Spine, and preexisting medical conditions.

**Conclusion:**

sSAE rates after treatment of sCS-Fx are high. The identified risk factors are not perioperatively modifiable, but their knowledge should guide intra and postoperative care and surgical technique.

## Introduction

About 2–3% of blunt trauma patients suffer injuries of the cervical spine, most frequently affecting the subaxial cervical spine (sCS) [1, 2]. sCS injury (sCS-Fx) is potentially devastating, with instability or cervical spinal cord injury (SCI) often necessitating surgical therapy [3] in the form of internal fixation, often including decompression [4, 5]. This, in turn, is associated with a substantial risk of adverse events (AEs) and especially surgical adverse events (SAEs), which can negatively influence neurological recovery, length of hospital stay, and increase treatment cost [6].

The literature on AEs in spine surgery is limited by the fact that many studies, while methodologically sound, analyze heterogenous patient samples including multiple anatomical regions of the spine and multiple pathologies, including neoplasia, degeneration, deformity, infection, and trauma. Consequently, studies report AE rates of 4 to over 80% [7–10], depending on patient population and study design. Severe SAEs requiring revision surgery in particular reportedly occur in up to 16% of patients [10–12] in any region of the spine treated for any indication. Few studies focus on AEs after treatment of specific pathologies in specific regions of the spine.

There are indications that sCS-Fx patients might be at higher risk for SAEs than patients treated electively, especially for degenerative disease [6, 12, 13]. This is plausible, since sCS-Fx patients present with a set of characteristic potential risk factors: There is no time for diligent preoperative planning or medical optimization in the face of acute instability or spinal cord compression [4, 5]. Multiple trauma is present in 40–53% of patients with sCS-Fx [1, 3], possibly complicating surgical treatment and postoperative care. Injury patterns are diverse, and preexisting spinal pathologies can further hamper standardized surgical treatment [14–16]. The influence of many of these potential risk factors, especially multiple trauma, injury morphology, fixation procedures, and preexisting pathologies of the spine, is not sufficiently clear.

The aim of this study is to determine the types and incidences of severe SAEs, as well as risk factors after operative treatment of traumatic injuries of the sCS in a cohort of consecutive patients.

## Methods

This is a single-center retrospective cohort study. It was conducted at a national trauma center and was approved by the ethics committee in charge (Ethics Committee of the State Medical Association Rhineland-Palatinate, Mainz, Germany; application number 2021–15816). The STROBE statement was followed for reporting of this study.

### Participants

#### Inclusion criteria

We included patients who fulfilled the following criteria in the detailed retrospective data collection: (1) underwent surgery for an acute discoligamentous injury and/or fracture of the sCS (vertebrae C3 to Th1, segments C3/4 to C7/Th1) at the study clinic from 2010 to 2020, (2) had a preoperative CT scan available for detailed injury classification, and (3) had no previous surgery of their sCS.

Patients with instability of the C2/C3 segment and no other fracture or discoligamentous injury of the sCS were not included.

#### Exclusion criteria

After detailed retrospective data collection, patients were excluded from the final analysis of SAEs if they were transferred to a different hospital or died within 14 days after operative treatment, if death did not occur as a direct consequence of surgery, and if the patient had not experienced an SAE up to that point. This was done to reduce the risk of bias due to non-recorded SAE, as we could not be sure these patients would not have developed an SAE after their transferal or death. Patients who died or were transferred within 14 days and had experienced an SAE were included in the analysis.

### Treatment

If after the initial CT scan injury morphology was not clear or neurological deficits were not sufficiently explained by CT findings, an MRI was performed. The decision of surgical treatment was made by the surgeon in charge and, if possible, in agreement with the patient or their family. Treatment decisions were based on recommendations in contemporary versions of international guidelines [4, 5], taking into consideration injury morphology, spinal cord compression, pre-existing conditions of the spine, concomitant injuries, and patients’ cardiopulmonary stability. Treatment methods comprised (i) a standard posterior approach with pedicle or lateral mass screws and internal rod fixation, as well as decompression if needed, and (ii) a standard anterior approach with discectomy and fusion. The anterior fusion was performed using an autologous iliac crest craft regularly up to 2015. From 2015 on, this was steadily replaced by intervertebral cages which were exclusively used from 2018 onward. Some patients received (iii) a combined anterior and posterior stabilization. Routinely, an intravenous perioperative single-shot antibiotic prophylaxis was used and repeated in case of ongoing surgery after 180 min. The decision on postoperative treatment with or without a cervical collar was made for each patient individually by the treating surgeon. Postoperative radiological controls were done with CT scans. All patients were recommended to undergo clinical and radiological follow-up 6 weeks postoperative at the study site or externally to detect possible secondary instability, dislocation, or implant loosening with a recommendation of referral back to our site in case of suspicious findings.

### Variables

#### Baseline characteristics

The following baseline characteristics were collected for all patients from the hospital’s database. Patient characteristics: age at injury, sex, preexisting medical conditions, use of direct oral anticoagulants or antiplatelet medication (DOAC or APM, yes/no), preexisting pathologies of the cervical spine (ankylosing spondylitis (AS), diffuse idiopathic skeletal hyperostosis (DISH), ossification of ligamentum flavum (OLF) or the posterior longitudinal ligament (OPLL), one or more present yes/no). The Charlson Comorbidity Index (CCI) [17] was calculated. Concomitant injuries including the presence of complete or incomplete SCI and Glasgow Coma Scale (GCS) were collected, and the Injury Severity Score (ISS) was calculated. Details of all surgical procedures of the sCS were collected: duration from injury to first surgery (days) and primarily intended surgical approach (anterior, posterior, combined) were documented. As the primarily intended surgical approach, we considered the documented initial treatment plan for a patient, meaning that in case of dislocation or persisting instability after the intended singular anterior or posterior approach, we counted this a severe SAE and the escalation to combined stabilization as revision surgery. The total duration of primary surgery of the cervical spine was calculated; if the primary treatment plan comprised two operations, then the sum of surgical times was calculated.

All patients’ injuries were classified according to the AO Spine Subaxial Injury Classification System (A/B/C) as well as neurological classification (N0, neurology intact; N1, transient neurological deficit; N2, radicular symptoms; N3, incomplete SCI; N4, complete SCI; NX, cannot be examined) [14, 18]. We applied the corresponding classification of facet injuries and of modifiers, which describe case-specific conditions possibly influencing treatment decisions (M1, posterior capsuloligamentous complex injury without complete disruption; M2, critical disc herniation; M3, stiffening bone disease; M4, vertebral artery abnormality or injury, one or more present yes/no). Facet injury status was also stratified concerning stability according to the AO Spine Subaxial Injury Classification: Here, F2 injuries are considered potentially unstable, while F3 and F4 injuries are considered unstable. We also evaluated the presence of multilevel primary injury and secondary injury (yes/no).

#### Definition and grading of surgical adverse events

As suggested in the literature [19], we defined an AE as an unexpected or undesirable event related to patient treatment. We opted against the term “complication” as it has been characterized as having consequences on patient outcome [19], which we could not safely evaluate in this retrospective design. As SAE, we defined any AE that was a direct consequence of the surgical procedure to the sCS and was not in accordance with the desired or expected postoperative course. We did not include medical AEs, such as myocardial infarction or venous thrombosis. SAEs were graded according to the system of Clavien and Dindo [20]. The primary endpoint for this study was the occurrence of one or multiple severe SAEs (sSAE, Clavien-Dindo grade III or higher [21]) within 60 days after surgery as documented during the initial hospital stay, during any readmissions or follow-up visits. These sSAEs are characterized by the need of surgical, radiological, or endoscopic intervention (Grade III), life-threatening complications requiring ICU management (Grade IV), or death (Grade V). Since our population included patients with multiple trauma, we did not choose Grade I and II SAE, such as pronounced postoperative pain or anemia, as endpoints for the analysis, as these can frequently not be directly attributed to the sCS-Fx and surgery. The 60-days window was chosen to also include revisions because of failure of the initial treatment plan in the form of instability, dislocation, and implant loosening detected in radiological follow-ups 6 weeks postoperative.

### Statistics

Statistical analyses were done with SPSS for Windows (Version 27, IBM Corp, Armonk, NY, USA) and “R” (Ver 4.3.0 The Free Software Foundation Inc. Boston, MA, USA). Probability values < 0.05 were considered significant. No adjustment for multiple testing was done in this exploratory analysis.

#### Incidence of severe surgical adverse events

The incidence of sSAEs in the patient population was reported as absolute numbers and as percentages of the total population. For the calculation of the incidence of sSAEs, all documented sSAEs were counted. However, recurrent sSAEs of the same kind (e.g., recurring wound healing disorder) in the same patient were only counted once.

#### Analysis of potential risk factors

For analysis of potential risk factors, patients were first divided into a group with sSAE and a group without sSAE. Various patient, injury, and treatment characteristics between the groups were compared.

For continuous risk factors, differences of group means were tested for significance with a two-sided *t*-test; for significant results, 95% confidence intervals (CI) were reported. Ordinal potential risk factors were tested for significance using the Mann–Whitney U test. Categorical potential risk factors were tested using the Chi-squared test. As an exception, the contingency table for the presence of multilevel primary injury was tested using Boschloo’s test [22] because of the low expected frequency of observations of multilevel primary injury. Patients with missing information regarding certain potential risk factors (e.g., unclear date of injury) were excluded from the analysis for the affected risk factor, which was separately reported for each analysis in the results section.

A binary logistic regression model for the occurrence of one or more sSAEs was created. The variables were selected using “Best Subset Selection.” The “Akaike Information Criterion” (AIC) was used as a selection criterion [23], whereby the optimal variable constellation for the model with the lowest AIC was assumed, resulting in the inclusion of the variables age, use of APM or DOACs, presence of an AO Spine modifier, and presence of a potentially unstable or unstable facet injury in the model. Covariates were tested for collinearity, and a receiver operating characteristic (ROC) curve analysis was performed for the model. For this calculation, “R” (Ver 4.3.0 The Free Software Foundation Inc. Boston, MA, USA) and the package “bestglm” were used. Odds ratios (OR) and 95% CIs were reported.

## Results

### Patient, injury, and treatment characteristics

A total of 319 patients were included in the detailed retrospective data collection. Of these, twelve patients were excluded from the final analysis of SAEs because of transferal to a different hospital within 14 days after surgery and 15 patients because of death within 14 days after surgery. None of these patients had a documented sSAE, and none of the deaths could be directly attributed to the sCS surgery, thus not meeting the criteria for sSAE as defined as this study’s primary endpoint. Reasons of death for the 15 excluded patients were cardiopulmonary failure attributed to SCI (*n* = 10), surgery-unrelated septic shock (*n* = 2), traumatic brain injury (*n* = 1), and advanced glioblastoma (*n* = 1) and prostatic cancer (*n* = 1) with pre-injury palliative care.

**Fig. 1:**
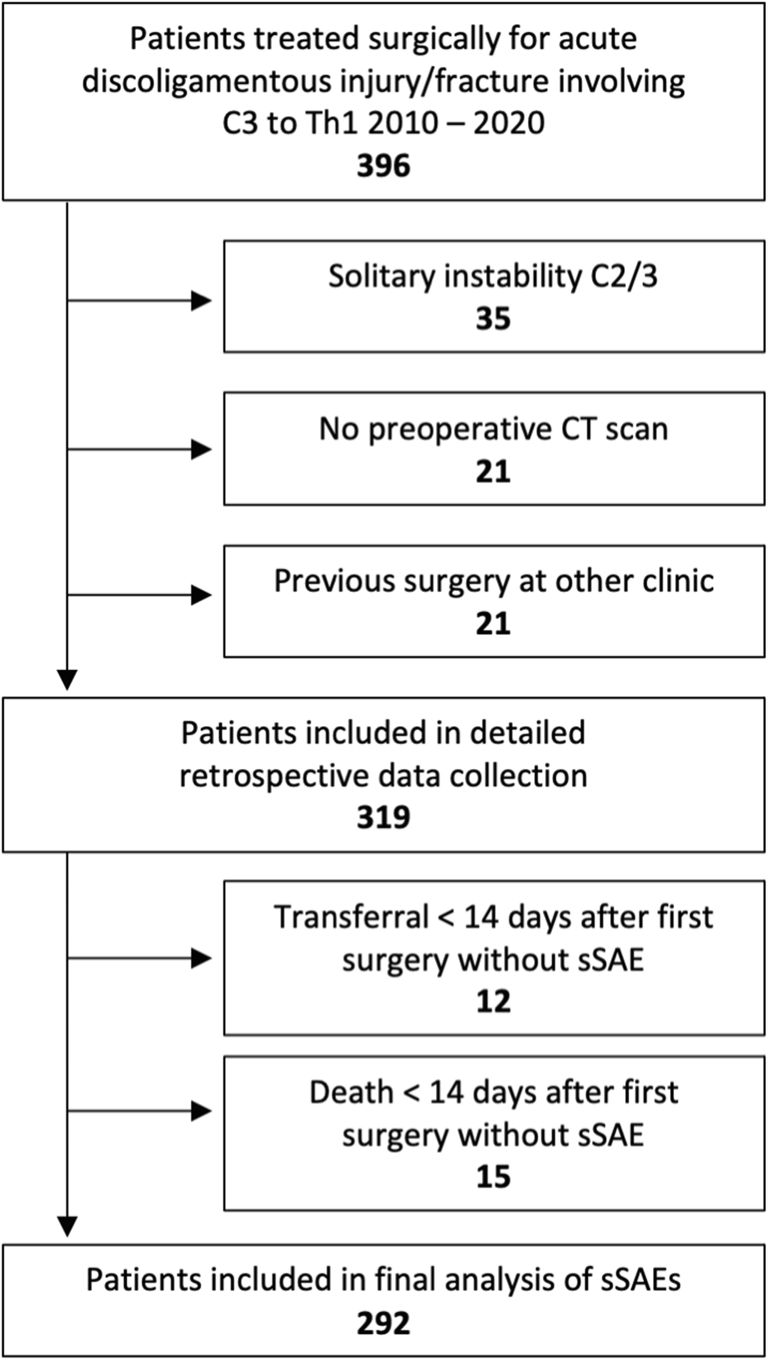
Details on the screening and inclusion process of patients included in the detailed retrospective data collection and the final analysis of severe surgical adverse events (sSAEs)

A total of 292 patients were included in the final analysis. Figure 1 details the application of inclusion and exclusion criteria. The mean follow-up was 198 days (median 95, range 6–1923 days), and 117 of the included patients (40.1%) had follow-up of less than 60 days (mean 32 days, median 29 days).

Patient demographics, comorbidities, and concomitant injuries are shown in Table 1. Mean age of patients was 57.8 years (SD 20.5, range 12–91 years). Median CCI was 0 (IQR 1, range 0–7), and median ISS was 13.5 (IQR 16.25, range 4–75). One hundred and forty-one patients (48.3%) had an ISS of at least 16 and were polytraumatized by this definition. Median GCS upon admission was 15 (IQR 0, range 3–15, seven patients not testable).

**Table 1:** Patient demographics, comorbidities, and concomitant injuries

Demographics, comorbidities				Concomitant injuries			
Sex	Male	217	74.3%	ISS	4–8	60	20.5%
	Female	75	25.7%		9–15	91	31.2%
Age	≤ 19	7	2.4%		16–24	18	6.2%
	20–39	55	18.8%		> 24	123	42.1%
	40–59	78	26.7%	GCS	13–15	249	85.3%
	60–79	112	38.4%		9–12	9	3.1%
	≥ 80	40	13.7%		3–8	27	9.2%
CCI	0	182	62.3%		nt	7	2.4%
	1–2	67	22.9%	Surgery for other injuries	No	211	72.3%
	3–4	30	10.3%		Yes	81	27.7%
	≥ 5	13	4.5%				
APM/DOACs^1^	No	234	80.7%				
	Yes	56	19.3%				
Preexisting spine pathology (AS, DISH, OLF, OPLL)	No	257	88.6%				
	Yes	35	12.1%				

*APM/DOACs*, antiplatelet medication/direct oral anticoagulants; *nt*, not testable; *AS*, ankylosing spondylitis; *DISH*, diffuse idiopathic skeletal hyperostosis; *OLF*, ossification of ligamentum flavum; *OPLL*, ossification of posterior longitudinal ligament

^1^Two patients with missing information

AO Spine Injury Classification, SCI classification, and treatment characteristics are shown in Table 2. Neurological classifications according to AO Spine were as follows: N0: 123 (42.1%), N1: 15 (5.1%), N2: 45 (15.4%), N3: 65 (22.3%), N4: 40 (13.7%), and NX: 4 (1.4%). One hundred and sixty patients (54.8%) had facet injuries classified as potentially unstable (type F2) or unstable (types F3 and F4). Median time from injury to surgery was 2 days (mean 4.1 days, SD 7.7, range 0–86 days); in two patients, the exact date of injury was unknown. Mean total duration of surgery for primary treatment of the subaxial cervical spine injury was 158.5 min (median 130.0 min, SD 92.9, range 45–468 min); 51 patients were not evaluable in this regard because documentation of surgical times did not clearly differentiate between treatment of the sCS and other injuries which were done in one session.

**Table 2:** Details on classification of cervical spine injury, neurological injury, and surgical procedures

AO Spine Injury Classification				Neurological injury			
Primary	A	17	5.8%	Spinal cord injury	None	183	62.7%
	B	135	46.2%		Incomplete	65	22.3%
	C	140	47.9%		Complete	40	13.7%
Secondary injury	None	131	44.9%		Not testable	4	1.4%
	A0	83	28.4%				
	A1	27	9.2%	Operations			
	A2	3	1.0%				
	A3	17	5.8%	Primary surgical approach	Anterior	134	45.9%
	A4	26	8.9%		Posterior	47	16.1%
	B2	2	0.7%		Combined	111	38.0%
	B3	3	1.0%	Time from injury to first surgery (days)^2^	< 1	87	30.0%
	No	272	93.2%		1	56	19.3%
	Yes	20	6.8%		2	28	9.7%
	None	102	34.9%		3–4	29	10.0%
	F1	30	10.3%		5–7	37	12.8%
	F2	44	15.1%		8–14	42	14.5%
	F3	16	5.5%		> 14	11	3.8%
	F4	100	34.2%	Duration of total surgery (min)^3^	< 90	70	24.0%
Modifier^1^	None	153	52.4%		90–119	40	13.7%
	1	100	34.3%		120–179	49	16.8%
	2	38	13.0%		180–239	34	11.6%
	3	35	12.0%		240–300	23	7.9%
	4	10	3.4%		> 300	25	8.6%

^1^More than one per patient possible. AO Spine Modifiers: M1, posterior capsuloligamentous complex injury without complete disruption; M2, critical disc herniation; M3, stiffening bone disease; M4, vertebral artery abnormality. ^2^Two missing: exact date of injury unknown. ^3^51 excluded: no demarcation to other surgical procedure

### Incidence of severe surgical adverse events

Forty-nine patients (16.8%) suffered at least one sSAE. Table 3 shows the frequency and incidence of different categories of sSAE. sSAE were graded as Clavien-Dindo grade III in 42 patients (14.4%) and grade IV in seven patients (2.4%).

**Table 3:** Type and incidence of severe surgical adverse events in the total sample (*n* = 292) within 60 days postoperatively

Severe surgical adverse event	Incidence	
	*n*	%
Reduction, implants, fixation	16	5.5%
Dislocation	11	3.8%
Implant loosening without dislocation	3	1.0%
Implant malposition	2	0.7%
Surgical site	29	9.9%
Wound healing disorder/infection	15	5.1%
Hematoma	7	2.4%
Seroma	6	2.1%
Edema/swelling	4	1.4%
CSF leakage	2	0.7%
Neurology	3	1.0%
Symptomatic recurrent nerve palsy	1	0.3%
Postoperative radiculopathy	1	0.3%
SCI deterioration	1	0.3%
Iliac crest bone graft harvesting	6	2.1%
Surgical site	5	1.7%
Meralgia paraesthetica	1	0.3%
Dysphagia	5	1.7%
Pneumothorax	1	0.3%

*CSF*, cerebrospinal fluid; *SCI*, spinal cord injury

The listed adverse events necessitated revision surgery, treatment in the ICU, or both (Clavien-Dindo grades III and IV). The number of adverse events is greater than the number of patients with adverse events, as some patients experienced more than one adverse event. The number for the category “surgical site” is lower than the sum of its subcategories because some patients experienced more than one adverse event related to their surgical site

### Potential risk factors for severe surgical adverse events

Table 4 shows the univariate analysis of factors potentially favoring the occurrence of sSAE with differences between the patient group with sSAE and the group without sSAE. Analysis of patient characteristics showed a statistically significant difference in mean age between the two groups: The group with sSAE was 8.9 years older (*p* = 0.002, 95% CI 3.6–16.1 years). Patients with sSAE had preexisting spine pathologies significantly more often (20.4% vs. 10.3%, *p* = 0.047). Sex (*p* = 0.621), severity of comorbidities measured by CCI (*p* = 0.347), and use of DOACs or APM (*p* = 0.058) were not significantly different between the groups.

**Table 4:** Potential risk factors for severe surgical adverse events after treatment of injuries to the subaxial cervical spine and their differences in patients with and without severe surgical adverse events

Potential risk factors	*n*	no sSAE	sSAE	*p*
Patient demographics and comorbidities				
Age [years, mean (SD)]	292	56.1 (20.5)	65.0 (18.7)	.002^1^
Sex [% female]	292	25.1	28.6	.612^2^
CCI [median (IQR)]	292	0 (1)	0 (2)	.347^3^
APM/DOACs	290	17.4%	29.2%	.058^2^
Preexisting spine pathology	292	10.3%	20.4%	.047^2^
Spinal cord and concomitant injuries				
ISS [median (IQR)]	292	11 (16)	14 (17)	.711^3^
GCS [median (IQR)]	285	15 (0)	15 (1)	.715^3^
SCI present	288	29.4%	40.8%	.487^2^
Surgical procedures				
Time from injury to first surgery [days, mean (SD)]	290	4.0 (6.2)	4.5 (12.6)	.720^1^
Duration total surgery [minutes, mean (SD)]	246	159.1 (76.1)	155.6 (96.1)	.828^1^
Primary surgical approach anterior/posterior/combined [%]	292	49.0/14.0/37.0	30.6/26.5/42.9	.025^2^
Required surgery for other injuries	292	28.8%	22.4%	.365^2^
AO Spine Subaxial Injury Classification				
Injury type A/B/C [%]	292	6.2/46.1/47.7	4.1/46.9/49.0	.850^2^
Multilevel primary injury present	292	7.4%	4.1%	.485^4^
Secondary injury present	292	53.5%	63.3%	.210^2^
Modifier present	292	44.4%	63.3%	.016^2^
FI type [median (IQR)]	292	2 (4)	4 (4)	.038^3^
(Potentially) unstable FI	292	52.7%	65.3%	.105^2^

*sSAE*, severe surgical adverse event; *SD*, standard deviation; *CCI*, Charlson Comorbidity Index; *IQR*, inter quartile range; *APM/DOACs*, antiplatelet medication/direct oral anticoagulants; *ISS*, Injury Severity Score; *GCS*, Glasgow Coma Scale; *SCI*, spinal cord injury; *FI*, facet injury. Statistical tests used: ^1^Two-sided *t*-test, ^2^chi-squared-test, ^3^Mann-Whitney U test, ^4^Boschloo’s test

A total of 40.8% of patients with sSAE initially presented with SCI compared to 29.4% of patients without sSAE. This difference was not statistically significant (*p* = 0.487). ISS (*p* = 0.711) as well as GCS (*p* = 0.715) were not significantly different. Four patients could not be evaluated for SCI and seven patients for GCS and were thus excluded from the corresponding analysis.

The primary surgical approach was significantly different between the groups (*p* = 0.025) with patients with sSAE having a posterior or combined approach in 69.4% compared to only 51.0% in the group without sSAE, thus making sSAE significantly less frequent if a solitary anterior primary approach was used. Time from injury to surgery (*p* = 0.720) and duration of surgical therapy (*p* = 0.946) were not statistically different. Two patients with unclear time from injury to surgery and 51 patients with unclear total surgical duration were excluded from the corresponding analysis. Patients with sSAE had surgery for other injuries less often (22.4% vs. 28.5%), yet without statistical significance (*p* = 0.386).

A comparison of detailed AO Spine Classification of the groups revealed that patients with sSAE had modifiers marking injury complexity significantly more often (63.3%) than patients without sSAE (44.4%; *p* = 0.016). They also had significantly higher mean ranks of concomitant facet injuries (*p* = 0.038). A non-significant trend towards the presence of potentially unstable or unstable facet injuries was present (*p* = 0.105). The other AO Spine parameters, primary injury A, B, or C (*p* = 0.850), presence of multilevel primary injury (*p* = 0.485), and presence of secondary injury (*p* = 0.210), were not significantly different. However, a secondary injury was markedly more prevalent in the group with sSAE (63.3% vs. 52.9%).

**Fig. 2:**
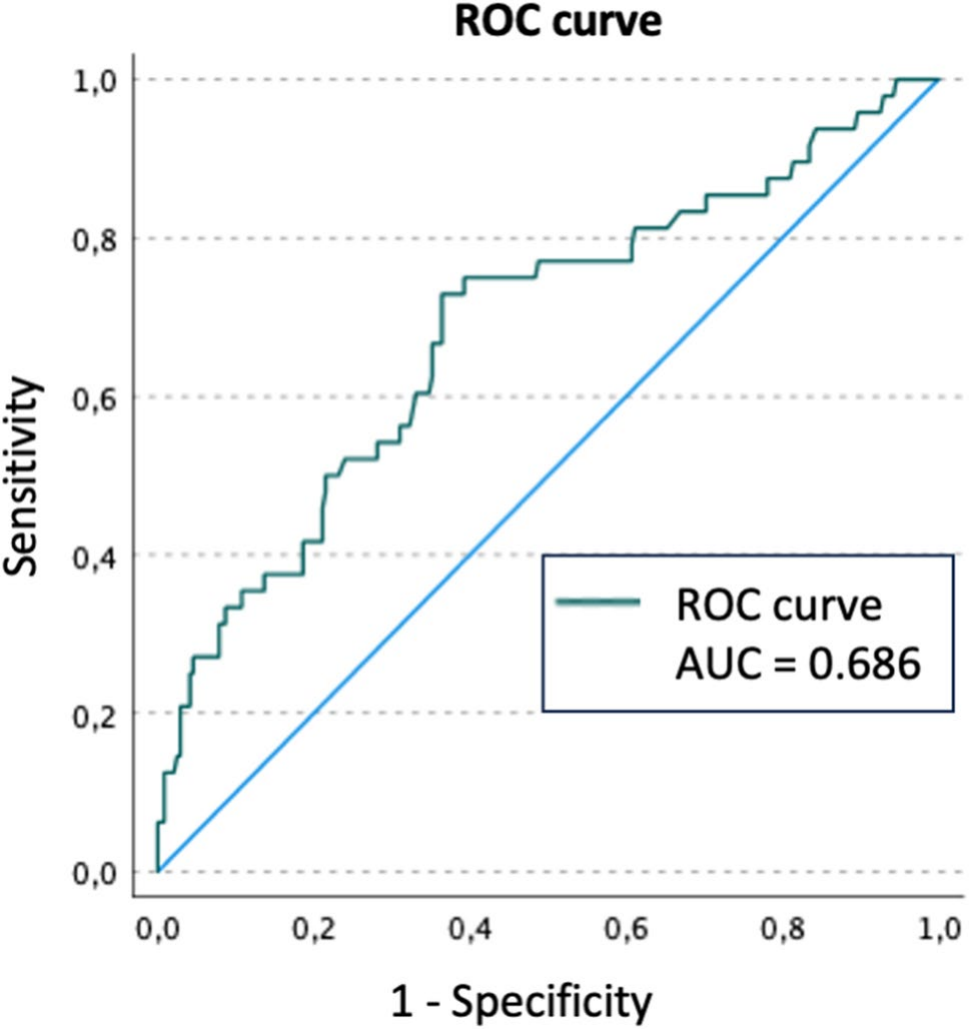
Receiver operating characteristic (ROC) curve of the binary logistic regression model for the occurrence of one or more severe surgical adverse events after treatment of injuries to the subaxial cervical spine. AUC, area under the curve

Variable selection for a binary logistic regression model for the occurrence of one or more sSAEs delivered the lowest AIC (248.7489) with the variables age, use of APM or DOACs, presence of an AO Spine modifier, and presence of a potentially unstable or unstable facet injury. The variable “duration of total surgery” was not included in the variable selection process because of the high number of missing values. The variables “type of facet injury” and "AO Spine Neurology” were also omitted, as high collinearity with the variables “(potentially) unstable facet injury” and “spinal cord injury,” respectively, which were included, was existent. The area under the ROC curve was 0.686, corresponding to a poor predictive value (< 0.7, Fig. 2).

In this model, statistically significant variables were age (*p* = 0.022), with a 2% (95% CI 0.3%–4.0%) increase per year of the odds of developing an sSAE, presence of an AO Spine modifier (*p* = 0.041), and presence of a potentially unstable or unstable facet injury (*p* = 0.010). Details are given in Table 5.

**Table 5:** Binary logistic regression model for the occurrence of one or more severe surgical adverse events after treatment of injuries to the subaxial cervical spine. Best subset selection, variable selection with “Akaike Information Criterion” (AIC) (21)

Variables	Coefficient	Odds ratio (95% CI)	*p*
Age (per 1 year increase)	0.023	1.02 (1.003–1.04)	.022
APM/DOACs	0.294	1.34 (0.60–3.01)	.476
AO Spine modifier	0.703	2.02 (1.03–3.96)	.041
Unstable facet injury	0.913	2.49 (1.24–4.99)	.010

*AIC*, Akaike Information Criterion; *ROC AUC*, receiver operating characteristic area under the curve; *CI*, confidence interval; *APM/DOACs*, antiplatelet medication/direct oral anticoagulants

Statistical model parameters: AIC = 248.7489; ROC AUC = 0.686

## Discussion

This single-center retrospective cohort study of 292 patients undergoing surgery for sCS-Fx found an incidence of sSAEs of 16.8% within 60 days after surgery, most frequent being sSAEs of the surgical site (wound healing disorder, infection, hematoma, etc.), followed by implant and fixation-related sSAE (dislocation, implant loosening, etc.). Independent potential risk factors in a binary logistic regression model were higher age, the presence of one or more modifiers in the AO Spine classification, and the presence of a potentially unstable or unstable facet injury. Other suspected risk factors were not statistically significant, among these ISS and the need for surgery for concomitant injuries, the duration of primary surgery, the primary injury type according to AO Spine, preexisting medical conditions measured by CCI, and the use of APM or DOACs. The primary surgical approach was a significant risk factor in univariate analysis but not in the multivariate logistic regression model.

### Defining adverse events in spine surgery

An aspect of paramount importance in any analysis of perioperative morbidity is the definition of what constitutes an AE in the first place. This question has been the center of much professional discussion in and of itself, and there is no consistent definition of the terms “complication” or “adverse event” in spine surgery which are also often used interchangeably [7, 24].

A disambiguation was suggested by Rampersaud et al. [19] who proposed that an “adverse event” is any unexpected or undesirable event related to patient treatment. An AE could possibly lead to a “complication,” which is in turn characterized by clinically significant sequelae and thus a negative impact on patient outcome. One could deduce that, therefore, without considering patient-reported outcome, only the analysis of “adverse events” and not of “complications” is viable in retrospective analysis. Thus, we opted for the term “adverse event” to describe our retrospectively analyzed endpoint of surgical morbidity.

It is also important to clarify which categories of AEs are taken into consideration as primary endpoint. We chose not to analyze indirect AEs such as pressure ulcers or medical adverse events like urinary tract infections or thromboembolic events, the reason being that these can rarely be attributed to a single injury or surgical procedure in the setting of trauma and especially multiple trauma, which in turn is often present in patients with sCS-Fx [3]. Instead, we chose to evaluate events with a clear causal relationship to the cervical spine procedure. Our intent in this was to give surgeons information on AEs that can directly be influenced by their decision making and surgical technique. Additionally, the grading of AEs applied here, according to direct treatment consequences as suggested by Dindo et al. [20], limits documentation bias, as severe AEs necessitating intervention in the operating room or treatment in the ICU are generally well documented.

Of course, the fact that a narrower category of postoperative AEs was evaluated in this analysis must be taken into consideration when comparing results to other studies in the literature. It is also important to state that medical or indirect AEs after spine surgery should not be considered less relevant for the patient, as every AE for itself or combined with others can have a negative impact on patient outcome [9].

### Incidence of adverse events in spine surgery

In studies comprising the entire spine and any indication for surgery, rates for any AE vary greatly and are reported as 10–16% [8, 25, 26], 34–52% [10, 12, 27], or as high as 87% [9]. Rates of complications requiring revision surgery are reported as 3–16% [10–12]. Systematic literature reviews found complication rates of 4–19% depending on anatomical region and indication for surgery [7, 8].

Comparing incidences of AEs is difficult not only due to different definitions and categories of AEs as explained above, but also because of great internal heterogeneity in many of the above cited study samples, which include different anatomical regions of the spine as well as various indications for surgery. There is a lack of studies with homogenous samples dealing with one anatomical region of the spine and a single pathological category. Thus, only a limited number of recent studies report detailed rates of AEs after surgery of the cervical spine because of trauma.

In 2016, Fredø et al. [28] published a retrospective single-center cohort study of 303 patients treated surgically for sCS-Fx between 2002 and 2010. They reported a reoperation rate due to AEs of 7.3%. AEs were malalignment (2.6%), SCI deterioration (2.0%), infection (1.3%), fixation device failure (1.3%), implant malposition (1.0%), suboptimal decompression (0.7%), CSF leakage (0.7%), and hematoma (0.3%). New radiculopathy occurred in 1.3% of patients. Of note, patients with ankylosing spondylitis were excluded, and the average patient age was only 48 years, almost 10 years lower than in our study. Additionally, only 31.0% of patients were treated with a posterior or combined approach, compared to 54.1% of patients in our cohort. These might be contributing factors to the substantially lower rate of sSAEs in the study of Fredø et al. when compared to our rate of 16.8%.

A thorough analysis of the incidence of SAEs and their impact on patient outcome, length of stay, and treatment costs was published by Liebscher et al. in 2022 [6]. They retrospectively analyzed 165 patients who underwent surgery for acute traumatic cervical SCI at a single center, not differentiating between the upper cervical spine and sCS. At least one SAE occurred in 22.4% of patients, SAEs being mechanical instability (7.9%), insufficient decompression (6.7%), hardware malposition (4.2%), wound infection (2.4%), spinal cord compression (1.8%), hematoma (1.8%), retropharyngeal scarring (1.8%), and vessel injuries (1.2%). They found an association of SAEs with reduced probability of neurological recovery, increased length of ICU and total hospital stay, and higher treatment costs, highlighting the immediate negative effects of SAEs for patients, caregivers, and the healthcare system.

A number of studies on AEs focus on the cervical spine but include any indication for surgery [13, 29, 30]. Unfortunately, in these cohorts, trauma patients are underrepresented and frequently there is no subgroup analysis.

Campbell et al. [29] prospectively analyzed AEs within 30 days after cervical spine surgery in 119 patients, including 31 trauma patients (26.1%). AEs of any kind (surgical or medical) occurred in 44.5% of patients in the total sample. Among the AEs we would consider SAEs were dysphagia (10.1%), deep wound infection (8.4%), C5 palsy (1.7%), durotomy (1.7%), vocal cord paralysis (1.7%), epidural hematoma (0.8%), and implant malposition (0.8%). There was no subgroup analysis for trauma patients. While this study’s strength lies in its prospective design, it excluded patients unable to consent, which might have led to the exclusion of elderly patients, patients with traumatic brain injury, or need for prolonged mechanical ventilation, possibly leading to lower rates of AEs and limiting comparability to our sample.

A retrospective study by Harel et al. [30] included 251 patients undergoing cervical spine surgery for any indication, including 35 trauma patients (13.9%), who did not undergo subgroup analysis. AEs of any kind occurred in 10.8% of patients, SAEs by our definition being neurological deterioration (6.4%), wound infection (3.6%), dural tear (2.8%), leakage of cerebrospinal fluid (2.0%), implant malposition (1.2%), and postoperative hematoma (0.4%). Revision of surgery for any reason was performed in 5.2% of patients.

Another prospective analysis by Yadla et al. [13] looked at early postoperative AEs in 121 patients undergoing cervical spine surgery, including 31 trauma patients (25.6%). The incidence of major AEs of any kind was 18.2% in the total sample and 19.4% among trauma patients. Incidences of particular categories of SAEs were deep wound infection (9.1%), graft/hardware malposition (4.1%), durotomy (4.1%), pneumothorax (3.3%), vocal cord paralysis (2.5%), excessive blood loss (2.5%), swallowing dysfunction (2.5%), pseudomeningocoele (0.8%), and epidural hematoma (0.8%). Separate incidences for trauma patients were not given.

Summarizing the cited literature on cervical spine SAEs, we found incidences of SAEs that are mostly comparable to our findings. Surgical site infection rates range from 1.3 to 9.1% [6, 13, 28–30] and occurred in 5.1% in our cohort; postoperative hematoma, which we saw in 2.4%, was reported in 0.8–1.8% in the literature [6, 13, 28–30]. Fredø [28] and Liebscher [6] reported dislocation rates of 5.0 and 7.9%. compared to 3.7% in our study. Implant malposition, which we saw in 0.7%, ranges from 0.8 to 4.2% [6, 13, 28–30] in the literature. Leakage of cerebrospinal fluid and postoperative radiculopathy, which we observed in less than 1% each, are rare SAEs, which is also reflected in the literature. Iatrogenic pneumothorax, which we saw once (0.3%), was only reported by Yadla et al. with a rate of 3.3% [13].

### Risk factors for severe surgical adverse events

#### Patient characteristics

We identified age as an independent risk factor of sSAEs. In this study, patients with sSAE were 8.9 years older (*p* = 0.002) than those without sSAE with a 2% (95% CI 0.3–4.0%) increase per year of increasing age of the odds of developing an sSAE. To no surprise, this association of advanced age and AEs in spine surgery is reliably reproduced in the literature [6, 11, 12, 25, 26, 31]. This is of paramount importance, especially in the context of trauma care, as there is a large and further rising number of elderly patients with cervical spine injuries [3, 32].

We saw no difference in CCI between patients with and without sSAE. CCI was generally low in our cohort, with 85.2% of patients having two or less points, which could be the result of documentation bias and might limit the validity of CCI reporting in our study. Liebscher et al. also found comparably low CCI values and only a weak association of CCI and higher rates of SAE in cervical spine trauma patients with SCI [6]. Other authors demonstrated an association of ASA grade and risk for any AE in spine surgery [12, 26], although Whitmore et al. found that CCI was more likely than ASA grading to predict AE risk [33]. More research into risks for spinal surgical AEs in particular is needed to determine, whether SAE rate is in fact influenced by comorbidities, or whether the latter solely dispose for medical AEs after spine surgery. Further research is also needed on whether APM or DOACs increase the risk of SAEs in sCS-Fx. We saw a clear trend, as these were almost twice as frequent in patients with sSAE (17.4 vs. 29.2%), which was, however, not statistically significant in univariate analysis and the regression model. A significant difference was seen in the univariate analysis of preexisting stiffening conditions of the cervical spine, which correspond to the modifier M3 in the AO Spine Classification and will be discussed there.

#### Concomitant injuries

Concomitant injuries are present in 40–53% of patients with cervical spine fracture, common being head, chest, extremity, and face injuries [1, 3]. However, there has not been much research into the relationship between concomitant injuries, their treatment, and SAEs in sCS-Fx. One might suspect that multiple trauma and surgery for other injuries could have a negative impact on treatment outcomes of sCS surgery. Contrary to this suspicion, we saw only a small, non-significant difference with greater ISS in patients with sSAE and no difference in GCS. We do not know of other studies having investigated this relationship. Interestingly, surgery for other injuries was more frequent in patients without sCS sSAE. This could be since patients without sSAE were younger, and these younger patients, having suffered high energy trauma more often, presented with concomitant extremity injuries more frequently, which were also treated surgically more often than in older patients, for whom conservative therapy might have been chosen more frequently. A negative impact on sCS outcome in patients with surgery for other injuries was not seen. Unfortunately, the literature on the influence of multiple trauma on sSAE in spine surgery is limited, and further research is desirable, as our investigation does not add conclusive results in this regard.

We saw a non-significant trend of higher sSAE rates in patients with SCI (19.0% vs. 15.8% in patients without SCI). In the SCI injury cohort of Liebscher et al. [6], SAEs occurred in 22.4% of patients, being slightly higher. A higher risk for sSAEs in SCI patients can therefore be suspected, but greater patient numbers are necessary to further investigate this trend.

#### Surgical procedures

We saw higher rates of sSAE for patients with a primary posterior or combined approach, which was statistically significant in univariate analysis, but not in the binary logistic regression model. Of course, higher sSAE rates for posterior approaches have been frequently described in the literature [8, 11, 29, 30] and are often attributed to substantially less soft tissue, increasing the risk for infections, wound healing disorders, relevant hematoma, and the development of CSF leakage. Although formally a modifiable risk factor, the primary surgical approach to the sCS is often dictated by injury morphology since sufficient stability or neural decompression may often only be achieved via a posterior approach. In cases where a posterior approach is necessary, the spectrum of sSAEs and their incidence should guide intraoperative and postoperative management and care.

Longer surgical time, which is a known risk factor for AEs in spine surgery in general [25] and infection in particular [34], only showed a non-significant trend in our cohort.

#### Injury morphology

The AO Spine Subaxial Injury Classification System [14, 18] is accepted and broadly applied as a consistent method of communicating injury morphology in the sCS [35]. We wanted to see whether aspects of this classification correlated with the incidence of sSAEs, reflecting higher injury complexity and thus more challenging surgical treatment. While a marginal effect of primary injury classification in the cervical spine on SAE rate had previously been shown [6], we did not observe significant differences concerning primary, secondary, or multilevel primary injury, although the latter was non-significantly more frequent in patients with sSAE.

Modifiers in the AO Spine Classification are denoted by the addition of M1–M4 and have been incorporated into the classification to convey case-specific and potentially complexing conditions that may influence clinical decision making [15]. In this study, the presence of at least one such modifier was an independent risk factor for sSAEs with an OR of 2.02 (95% CI [1.03–3.96]). Additionally, we saw facet injuries of significantly higher grade in patients with sSAEs in univariate analysis. In our binary logistic regression model, potentially unstable or unstable facet injury (types F2 and higher) was an independent risk factor for sSAEs with an OR of 2.49 (95% CI [1.24–4.99]). Our data thus suggest a prognostic value of AO Spine modifiers as well as the grade of accompanying facet injuries and highlight the value of classifying sCS-Fx according to the AO Spine Classification. Further research into associations of specific modifiers and facet injuries with particular sSAEs could guide perioperative decision making and postoperative care.

#### Trauma as a risk factor

One question this study is unable to answer by design is whether trauma itself is a risk factor for sSAEs. There are, however, indications in the literature that trauma cases might be at higher risk for AEs than especially elective degenerative cases. Yadla et al., analyzing cervical spine procedures, found a slightly higher AE rate in trauma cases (19.4%) compared to degenerative cases (16.0%) without statistical significance [13]. For any anatomical region of the spine, Kimmel et al. found that emergency cases (not exclusively comprising trauma) were at significantly higher risk for any AE with an OR of 3.35 [25], while Coia et al. found higher ORs for severe AEs for trauma cases than in degenerative and oncologic cases, yet without statistical significance [12]. Another important aspect in this regard is that trauma usually necessitates fusion procedures, which are associated with AEs more strongly than simple decompression procedures [10, 25].

### Value of perioperative risk modelling in trauma

In our view, preoperative risk modelling plays a fundamentally different role in emergency settings than in degenerative disease. Surgical procedures for degenerative disorders are usually elective and can thus be postponed until further medical optimization is acquired, or the operation can be omitted entirely if perioperative risk is higher than the suspected benefit for the patient. Even in oncologic cases, preoperative risk modelling might, in palliative settings, lead to a decision against surgery. In contrast, acute instability or spinal cord compression resulting from trauma often requires immediate intervention, and conservative therapy is often not an option.

The goal of risk modelling in trauma should be to identify patients at high risk for AEs in order to guide intraoperative decisions and postoperative care. Possible consequences could be more intense nursing care, more cautious postoperative mobilization and radiological controls, or in specific cases more generous antibiotic prophylaxis. A better understanding of patient, treatment, and injury-related associations with specific SAEs could thus help reduce postoperative morbidity. Further research into, e.g., the association of perioperative bacteremia and surgical site infection or the relationship of surgical approach, implants, and bone quality to secondary instability and dislocation could be conducted by means of case–control studies.

### Limitations

Some important limitations must be considered. First, this is a retrospective single-center study with its known categories of bias. Site-specific as well as individual surgeons’ treatment preferences and protocols might influence sSAE rates, introduce bias, and limit the generalizability of our findings. It has also been shown that retrospective analysis of AEs is inferior to prospective studies, resulting in an underestimation of AE rates [9, 27]. However, this effect might be less pronounced in the cervical spine [8]. We attempted to further mitigate this limitation by choosing a generally well-documented primary endpoint, being surgical intervention or treatment in the ICU.

This is an exploratory analysis of potential risk factors, as such all statistically significant results must be interpreted cautiously as no adjustment for multiple testing was made. Some patients had to be excluded from certain factor analyses because of missing data, which is clearly stated in every instance in the results section. The binary logistic regression model yielded only a poor predictive value with an area under the ROC curve of 0.686 and must thus be interpreted very cautiously. This analysis could not include all possibly relevant risk factors due to insufficient documentation of some variables, e.g., body mass index or smoking status.

Some important limitations concerning follow-up must be pointed out: Twenty-seven patients were excluded from the analysis, because they died or were transferred to a different hospital within 14 days after surgery without experiencing an sSAE. Since none of these patients had any documented sSAEs during the observed time, their inclusion would have led to a lower and possibly underestimated rate of sSAEs, which we wanted to rule out. Beyond this, we did not define minimum follow-up durations as inclusion criteria. While all patients were recommended to undergo follow-up for at least 6 weeks postoperative and present to the study clinic in case of suspicious findings or adverse events, it is possible that some adverse events, especially relating to treatment plan failure, might have been treated elsewhere.

Inhomogeneity in our patient cohort must be pointed out: Patients from different age groups, with various injury morphologies and surgical treatment modalities, were included. We tried to overcome limitations due to inhomogeneity by applying validated classifications, scores, and grading systems. In comparison to many other studies on AEs and risk factors, the present study’s strength lies in the focus on traumatic injury and its, compared to most single-center studies, large patient sample.

## Conclusion

The rate of sSAE in sCS-Fx patients is 16.8%, leading categories being sSAEs of the surgical site (9.9%) and fixation and implant-related sSAE (5.5%). Independent potential risk factors are patient age, AO Spine modifiers, and potentially unstable or unstable facet injuries. Since these risk factors are not perioperatively modifiable, their knowledge should guide intra and postoperative care and surgical technique. Further analysis of homogenous patient samples to develop patient, injury, and treatment-specific risk profiles and prediction models in trauma patients is necessary.

## Abbreviations

AE: Adverse event
AIC: Akaike Information Criterion
APM: Antiplatelet medication
AS: Ankylosing spondylitis
CCI: Charlson Comorbidity Index
CI: Confidence interval
DISH: Diffuse idiopathic skeletal hyperostosis
DOAC: Direct oral anticoagulants
GCS: Glasgow Coma Scale
IQR: Inter-quartile range
ISS: Injury Severity Score
OLF: Ossification of ligamentum flavum
OPLL: Ossification of posterior longitudinal ligament
OR: Odds ratio
ROC: Receiver operating characteristic
SAE: Surgical adverse event
SCI: Spinal cord injury
sCS-Fx: Subaxial cervical spine injury
sCS: Subaxial cervical spine
SD: Standard deviation
